# Integrated endocrine–oxidative–metabolic and trace element responses to heat and cold stress in dromedary camels (*Camelus dromedarius*)

**DOI:** 10.1186/s12917-026-05866-1

**Published:** 2026-09-09

**Authors:** Mariam Gamal Zaki, Eman Shawky Ramadan, Mohamed Ibrahim Oraby, Noha Mahmoud Ryad

**Affiliations:** Department of Internal Medicine and Infectious Diseases, Faculty of Veterinary Medicine, Cairo University, Giza, 12211 Egypt

**Keywords:** Dromedary camel (*Camelus Dromedarius*), Heat stress, Cold stress, Oxidative stress, Hormones, Metabolic and trace minerals

## Abstract

**Background:**

The dromedary camel (*Camelus dromedarius*) possesses remarkable physiological adaptations to withstand harsh desert conditions, including heat stress and drought. This study aimed to evaluate the effects of heat and cold stress on oxidative stress biomarkers, hormones, lipid, and mineral concentrations in dromedary camels. A total of 45 apparently healthy adult male dromedary camels were included in the study. Blood samples were collected, plasma and serum were separated for the determination of oxidative stress biomarkers, hormones, metabolic, and trace mineral parameters.

**Results:**

During heat stress, cortisol, zinc, and copper concentrations increased significantly (*p* < 0.05), whereas total antioxidant capacity (TAC), triiodothyronine (T3), glucose, and triglyceride concentrations decreased significantly (*p* < 0.05). In contrast, cold stress was associated with significant (*p* < 0.05) increases in catalase (CAT), malondialdehyde (MDA), T3, glucose, and cortisol concentrations, while TAC, triglycerides, and copper concentrations decreased significantly (*p* < 0.05). Strong positive (*p* < 0.01) correlation between T3 and thyroxine (T4), whereas a strong negative (*p* < 0.01) correlation was observed between zinc and cholesterol. Positive (*p* < 0.05) correlations were also detected between glucose and MDA, cortisol and CAT, and copper and TAC. Conversely, negative (*p* < 0.05) correlations were found between triglycerides and CAT, triglycerides and T4, triglycerides and cortisol, zinc and triglycerides, cholesterol and glucose, and copper and MDA. During summer, positive correlations were observed between glucose and MDA, T3 and T4, as well as between cholesterol and both T3 and T4. In contrast, glucose showed negative correlations with both T3 and T4, while zinc was negatively correlated with copper. During autumn, positive correlations were detected between CAT and TAC, T3 and T4, and glucose and zinc. Conversely, negative correlations were observed between triglycerides and TAC, cortisol and T4, glucose and cholesterol, and zinc and cholesterol. During winter, T3 was positively correlated with zinc but negatively correlated with TAC.

**Conclusions:**

Cold stress exerted a more pronounced effect on oxidative balance and metabolic activity in dromedary camels than heat stress. Regular monitoring of these parameters is recommended for the early detection of physiological disturbances during the cold season.

## Background

Dromedary camel (*Camelus dromedarius*), commonly known as the one-humped camel, is a unique large animal belonging to the family Camelidae. It possesses remarkable physiological adaptations that enable it to survive under harsh desert conditions. These adaptations allow camels to regulate body temperature, conserve water, and protect cellular components against proteotoxic stress, thereby ensuring survival in arid regions characterized by high ambient temperatures [1]. Both heat and cold are important environmental stressors affecting animals inhabiting arid and semi-arid regions [2]. Seasonal heat and cold stress have been shown to markedly influence physiological responses, energy balance, and serum biochemical parameters in animals [3].

Hormones secreted by the adrenal and thyroid glands play essential roles in thermoregulation, metabolic adaptation, and reproduction in livestock species, including camels [4, 5]. In dromedary camels, thyroid and adrenal gland functions exhibit considerable adaptability to desert environments and are strongly influenced by seasonal changes [5].

Oxidative stress is defined as an imbalance between the production of reactive oxygen species (ROS) and the antioxidant defense system, leading to cellular and tissue damage. Excessive ROS production can adversely affect proteins, lipids, and nucleic acids [6]. Environmental stress conditions may markedly increase ROS generation in camels, thereby inducing oxidative stress and impairing antioxidant defenses, which may predispose animals to various health disorders [2, 7]. Moreover, the mitochondrial antioxidant defense system is closely associated with thyroid status [8]. Thyroid hormones have been reported to regulate the activities of antioxidant enzymes, including superoxide dismutase (SOD), CAT, and glutathione peroxidase (GPx), in lymphoid tissues and skeletal muscles [9].

Thyroid hormones are also important regulators of general metabolism. Their circulating concentrations are influenced by metabolic status, seasonal variation, and water availability [3, 9]. These hormones regulate energy metabolism, particularly carbohydrate and lipid metabolism [10]. Previous studies have demonstrated that extreme hot or cold conditions significantly affect lipid metabolism and serum lipid profiles in camels, possibly through thyroid hormone-mediated mechanisms [3].

Trace minerals such as zinc and copper are essential cofactors for several antioxidant enzymes [11]. Zinc acts as a potent antioxidant through scavenging free radicals and inhibiting ROS production via suppression of NADPH oxidase activity [12]. In addition, zinc stimulates the synthesis of metallothionein, a cysteine-rich compound with strong hydroxyl radical-scavenging properties, and enhances the activity of antioxidant molecules and enzymes such as glutathione and CAT [13]. Copper is another essential trace element involved in numerous biochemical pathways and serves as a cofactor or structural component of many metalloenzymes. Copper-zinc superoxide dismutase (Cu/Zn-SOD) represents one of the major antioxidant enzymes responsible for free radical scavenging [6].

Several previous studies have investigated oxidative stress biomarkers, hormonal profiles, and serum biochemical parameters in healthy camels and in camels exposed to heat stress [9, 10, 14]. Other studies have focused mainly on oxidative stress biomarkers alone or in combination with hormonal changes during seasonal variations [2, 3, 5]. However, to our knowledge, limited information is available regarding the combined effects of both heat and cold stress on oxidative stress biomarkers, hormonal profile, serum lipid profile, and trace mineral concentrations in dromedary camels. Therefore, the present study was designed to evaluate these parameters and investigate their interrelationships in dromedary camels under Egyptian environmental conditions.

## Materials and methods

### Animals and study design

The present study was conducted on 45 apparently healthy adult male dromedary camels (15 animals per season), with body weights ranging from 500 to 700 kg and ages ranging from 5 to 7 years. The animals were reared in private farms located in Cairo and Giza governorates, Egypt. The two farms are located within the Greater Cairo area, ensuring similar climatic conditions throughout the study period. All animals were fed dried alfalfa along with concentrates. Mineral blocks and fresh water were available ad libitum throughout the day. The two farms followed similar animal management systems and feeding regimes throughout the study period. All animals were maintained under a regular vaccination and deworming program. Prior to sample collection, each camel underwent a complete physical examination.

Samples were collected at the end of each season to ensure adequate exposure to the prevailing environmental conditions. Summer samples were collected in September 2025 after prolonged exposure to heat stress conditions, whereas winter samples were collected in February 2026 following exposure to cold stress conditions. Autumn was considered the thermoneutral season and represented the baseline condition; therefore, samples were collected in November 2025.

### Estimation of ambient temperature and humidity

Meteorological data, including average daily ambient temperature (T) and relative humidity (RH), were obtained from the Egyptian Meteorological Authority for Cairo and Giza governorates during the study period (June 2025 to February 2026). The temperature–humidity index (THI) was calculated according to [15] using the following equation: THI = (1.8 × T + 32) − [(0.55 − 0.0055 × RH) × (1.8 × T − 26)]. Based on THI values, camels were considered to be under thermoneutral conditions during autumn (October–December), heat stress conditions during summer (June–September), and cold stress conditions during winter (January–February).

### Sample collection and analysis

Blood samples were collected in the early morning without sedation and with minimal restraint. Samples were obtained by jugular venipuncture, divided into two tubes. The first was an EDTA tube used for plasma separation and subsequent determination of oxidative stress biomarkers. The second was a plain tube used for serum separation for hormonal, lipid, and trace mineral analyses. Samples were transported to the laboratory in an ice box for further processing.

For serum analysis, blood samples collected in plain tubes were centrifuged at 3000 rpm for 10 min to obtain serum. Serum samples were stored at − 20 °C until analysis. Serum glucose, triglycerides, cholesterol, zinc, and copper concentrations were determined using a Microlab 300 clinical chemistry analyzer (Netherlands) and commercial kits supplied by Spectrum company, Egypt. Serum hormone concentrations, including T3, T4, and cortisol, were measured using ELISA kits (Sunlong Biotech, China). Oxidative stress biomarkers, including CAT, TAC, and MDA, were determined in plasma using commercial kits supplied by Biodiagnostic Company, Egypt, and measured using an APEL spectrophotometer (Japan).

### Statistical analysis

Statistical analyses were performed using SPSS software version 25. Data normality was assessed using the Shapiro–Wilk test. Differences among seasons were analyzed using one-way analysis of variance (ANOVA), followed by Tukey’s honestly significant difference (HSD) post hoc test. Pearson’s correlation coefficient was used to evaluate the relationships among hormone concentrations, oxidative stress biomarkers, lipid profile, and trace mineral concentrations. Data are presented as mean ± standard error (SE), and differences were considered statistically significant at *P* < 0.05.

## Results

**Fig. 1:**
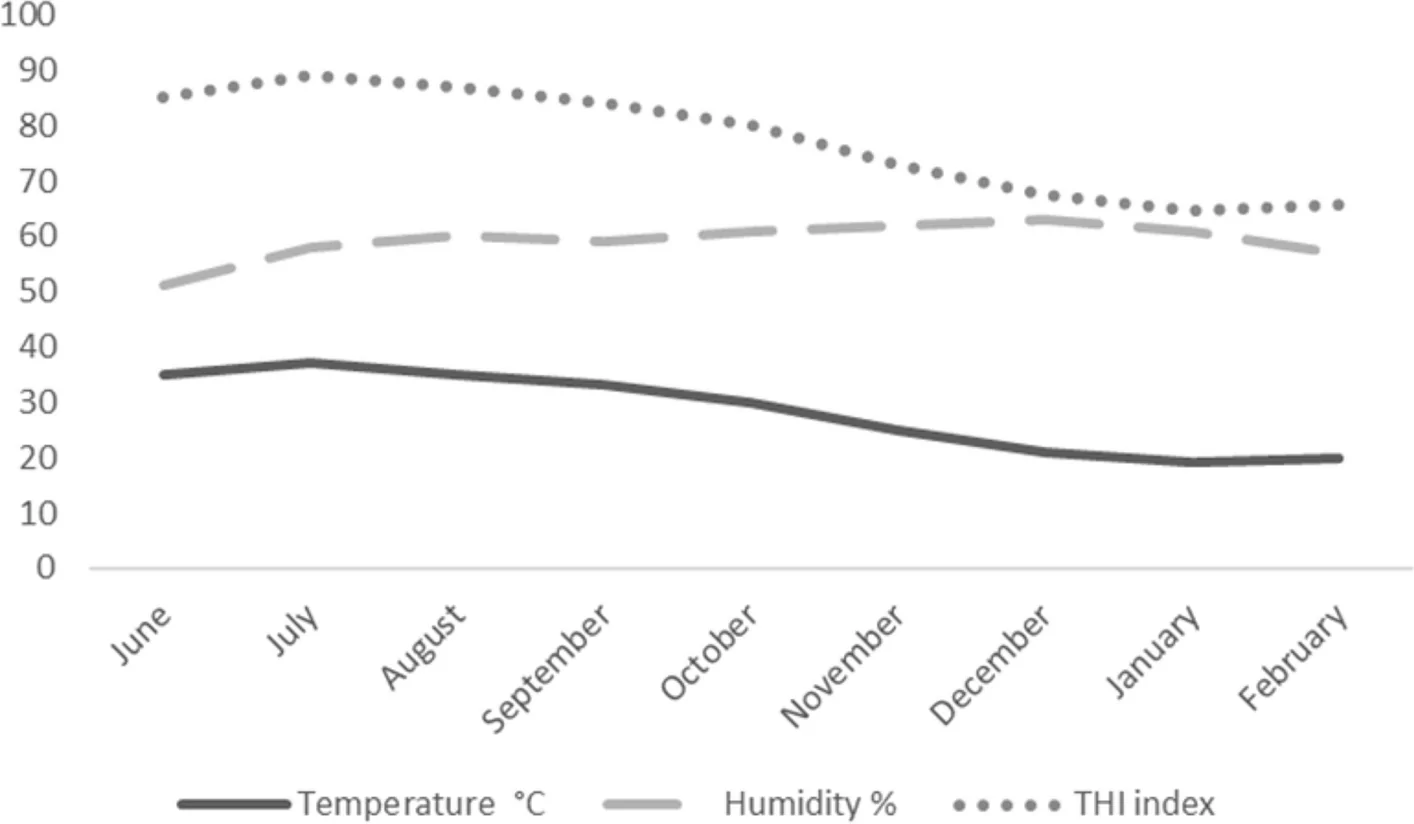
Temporal fluctuations in mean ambient temperature (°C), humidity (%) and temperature humidity index (THI) throughout the study period (from June 2025 to February 2026) in Giza and Cairo governorate (The Greater Cairo), Egypt

Ambient temperature and THI showed a gradual decline from summer to winter, while relative humidity exhibited only minor seasonal fluctuations (51%−61%) (Fig. 1). Summer recorded the highest mean ambient temperature (T = 37 °C) and THI (89.22) values, whereas winter showed the lowest values (T = 19 °C and THI = 64, respectively). In contrast, autumn was associated with intermediate environmental conditions (T = 25 °C and THI = 73, respectively).

The effects of heat and cold stress on oxidant antioxidant biomarkers, thyroid hormones, cortisol, lipids, and minerals, as well as the correlations among these parameters in dromedary camels reared under Egyptian conditions, are presented in Tables 1 and 2, respectively.

**Table 1:** Effect of heat and cold stress on hormones, oxidant antioxidant biomarkers, trace minerals and lipid profiles in dromedary camels

Parameters/unit	Summer group	Autumn group	Winter group
Catalase (U/L)	395.59 ± 43.02^b^	316.01 ± 26.71^b^	464.55 ± 59.55^a^
Malondialdehyde (nmol/ml)	1.29 ± 0.22 ^b^	0.92 ± 0.12 ^b^	3.16 ± 0.62 ^a^
Total antioxidant (Mm/L)	0.62 ± 0.14 ^b^	1.02 ± 0.06 ^a^	0.69 ± 0.01 ^b^
T3 (nmol/l)	2.33 ± 0.20 ^b^	2.36 ± 0.28 ^b^	3.96 ± 0.42 ^**a**^
T4 (nmol/l)	130.10 ± 10.94	113.21 ± 5.17	143.72 ± 9.27
Cortisol (nmol/l)	179.71 ± 4.30 ^a^	158.71 ± 6.47 ^**b**^	177.00 ± 7.13 ^a^
Glucose (mg/dl)	87.08 ± 10.26 ^b^	101.33 ± 9.80 ^b^	121.02 ± 6.16 ^**a**^
Cholesterol (mg/dl)	49.70 ± 5.25	57.44 ± 2.81	50.80 ± 1.76
Triglyceride (mg/dl)	76.37 ± 1.84 ^b^	109.70 ± 4.88 ^**a**^	68.56 ± 2.04 ^b^
Zinc (ug/dl)	104.06 ± 7.70 ^a^	63.41 ± 9.03 ^**b**^	75.82 ± 5.27 ^b^
Copper (ug/dl)	71.72 ± 9.32 ^a^	81.01 ± 2.13 ^a^	50.76 ± 6.13 ^**b**^

Data represented as mean ± SE, values in the same row having different superscript letters (^a^ and ^b^) are significantly different at *P* < 0.05

**Table 2:** Correlation between hormones, oxidant antioxidant biomarkers, minerals and lipid profiles in dromedary camels

	Catalase	Malonaldehyde	Total antioxidant	T3	T4	Glucose	Cortisol	Triglycerides		Cholesterol	Zinc	Copper
Catalase		*r* = 0.158 *p* = 0.606	*r* = −0.154 *p* = 0.569	*r* = 0.463 *p* = 0.071	*r* = 0.327 *p* = 0.216	*r* = 0.140 *p* = 0.606	*r* = 0.563* *p* = 0.023	*r* = −0.567* *p* = 0.022		*r* = 0.073 *p* = 0.789	*r* = 0.076 *p* = 0.780	*r* = −0.235 *p* = 0.381
Malondialdehyde			*r* = −0.292 *p* = 0.311	*r* = 0.458 *p* = 0.075	*r* = −0.008 *p* = 0.978	*r* = 0.597* *p* = 0.015	*r* = 0.042 *p* = 0.877	*r* = −0.397 *p* = 0.128		*r* = −0.303 *p* = 0.255	*r* = −0.021 *p* = 0.939	*r* = −0.564* *p* = 0.023
Total antioxidant				*r* = −0.025 *p* = 0.924	*r* = −0.166 *p* = 0.524	*r* = 0.007 *p* = 0.979	*r* = −0.369 *p* = 0.145	*r* = 0.467 *p* = 0.059		*r* = 0.404 *p* = 0.108	*r* = −0.389 *p* = 0.123	*r* = 0.515* *p* = 0.034
T3					*r* = 0.722** *p* = 0.000	*r* = 0.070 *p* = 0.776	*r* = −0.061 *p* = 0.805	*r* = −0.381 *p* = 0.108		*r* = 0.150 *p* = 0.539	*r* = −0.059 *p* = 0.811	*r* = −0.440 *p* = 0.059
T4						*r* = −0.389 *p* = 0.100	*r* = 0.140 *p* = 0.567	*r* = −0.469* *p* = 0.043	*r* = 0.356 *p* = 0.135		*r* = −0.001 *p* = 0.995	*r* = −0.225 *p* = 0.355
Glucose							*r* = 0.017 *p* = 0.945	*r* = −0.124 *p* = 0.614		*r* = −0.464* *p* = 0.045	*r* = 0.143 *p* = 0.560	*r* = −0.170 *p* = 0.487
Cortisol								*r* = −0.540* *p* = 0.017		*r* = −0.007 *p* = 0.978	*r* = 0.241 *p* = 0.320	*r* = −0.063 *p* = 0.797
triglycerides										*r* = 0.347 *p* = 0.146	*r* = −0.552* *p* = 0.014	*r* = 0.453 *p* = 0.052
Cholesterol											*r* = −0.634** *p* = 0.004	*r* = 0.294 *p* = 0.222
Zinc												*r* = −0.277 *p* = 0.251

Data are presented as Pearson correlation coefficient (r) and significance level (*p*-value)

^*^Correlation is significant at the 0.05 level (2-tailed)

^**^Correlation is significant at the 0.01 level (2-tailed)

Autumn was considered as a control season, representing baseline physiological conditions against which summer and winter variations were compared (Table 1). During the summer season (heat stress), cortisol, zinc, and copper showed significant (*p* < 0.05) increases, whereas TAC, T3, glucose, and triglycerides showed significant (*p* < 0.05) decreases. In autumn, TAC, triglycerides, and copper exhibited significant (*p* < 0.05) higher values, while CAT, malondialdehyde, T3, glucose, cortisol, and zinc showed significant (*p* < 0.05) lower values. In winter (cold stress), CAT, malondialdehyde, T3, glucose, and cortisol exhibited significant (*p* < 0.05) increases, whereas TAC, triglycerides, and copper showed significant (*p* < 0.05) decreases.

Significant correlations among thyroid and adrenal hormones, oxidant antioxidant biomarkers, minerals, and metabolic profiles in dromedary camels reared under Egyptian conditions are presented in Table 2. Very strong positive correlation was found between T3 and T4 (*r* = 0.722, *p* < 0.01), whereas a very strong negative correlation was detected between zinc and cholesterol (*r* = −0.634, *p* < 0.01). Positive correlations were observed between glucose and malondialdehyde (*r* = 0.597, *p* < 0.05), cortisol and CAT (*r* = 0.563, *p* < 0.05), and copper and TAC (*r* = 0.515, *p* < 0.05). Negative correlations were identified between triglycerides and CAT (*r* = −0.567, *p* < 0.05), triglycerides and T4 (*r* = −0.469, *p* < 0.05), triglycerides and cortisol (*r* = −0.540, *p* < 0.05), zinc and triglycerides (*r* = −0.552, *p* < 0.05), cholesterol and glucose (*r* = −0.464, *p* < 0.05), and between copper and malondialdehyde (*r* = −0.564, *p* < 0.05).

Seasonal correlation analysis revealed distinct correlation patterns among the studied parameters (Table 3). During summer, MDA showed a significant positive correlation with glucose (*r* = 0.781, *p* < 0.05). Thyroid hormones were strongly positively correlated (T3–T4, *r* = 0.987, *P* < 0.01), while both T3 and T4 exhibited significant (*P* < 0.01) negative correlations with glucose (*r* = − 0.889, and *r* = − 0.927, respectively). In addition, cholesterol was positively correlated with both T3 (*r* = 0.881, *P* < 0.01) and T4 (*r* = 0.837, *p* < 0.05), whereas zinc was negatively correlated with copper (*r* = − 0.771, *p* < 0.05). During autumn, CAT activity was positively correlated with TAC (*r* = 0.883, *P* < 0.01), whereas triglycerides showed a significant negative correlation with TAC (*r* = − 0.784, *p* < 0.05). A significant positive correlation was also observed between T3 and T4 (*r* = 0.777, *p* < 0.05). Cortisol was negatively correlated with T4 (*r* = − 0.815, *p* < 0.05). Furthermore, glucose showed a strong negative correlation with cholesterol (*r* = − 0.956, *P* < 0.01) and a positive correlation with zinc (*r* = 0.829, *p* < 0.05), while zinc was negatively correlated with cholesterol (*r* = − 0.861, *p* < 0.05). During winter, T3 exhibited a significant negative correlation with TAC (*r* = − 0.972, *p* < 0.05) and a significant positive correlation with zinc (*r* = 0.938, *p* < 0.05).

**Table 3:** Summary of significant correlations among hormones, oxidant antioxidant biomarkers, minerals, and lipid profile parameters during summer, autumn, and winter in dromedary camels

Season	Variables	Pearson correlation coefficient (r)	Significance level (*p*-value)
Summer	Glucose—Malondialdehyde	0.781	0.038
	T3-T4	0.987	0.000
	Glucose – T3	−0.889	0.007
	Glucose – T4	−0.927	0.003
	Cholesterol- T3	0.881	0.009
	Cholesterol- T4	0.837	0.019
	Zinc—copper	−0.771	0.043
Autumn	Catalase- total antioxidant capacity	0.883	0.008
	Triglycerides—total antioxidant capacity	−0.784	0.037
	T3-T4	0.777	0.040
	Cortisol – T4	−0.815	0.026
	Glucose- cholesterol	−0.956	0.001
	Glucose- zinc	0.829	0.021
	Zinc—cholesterol	−0.861	0.013
Winter	T3- total antioxidant capacity	−0.972	0.028
	T3- zinc	0.938	0.018

## Discussion

Extreme temperature is considered one of the major environmental stressors affecting animal physiology, health, production, and reproduction [16]. Unlike previous studies that primarily focused on oxidative stress and endocrine responses, the present study provides an integrated assessment of oxidative, endocrine, metabolic, and trace element responses to heat and cold stress in dromedary camels bred under Egyptian conditions.

The mean values of oxidative stress biomarkers, including CAT, MDA, and TAC, observed in this study were consistent with previous reports [2, 5]. CAT and MDA showed significant increases during the winter season compared to summer and autumn, in agreement with previous findings [5, 17]. The elevated levels of CAT and MDA may be attributed to increased free radical generation under stress conditions, leading to oxidative damage of the erythrocyte membrane [5]. The observed increase in CAT and MDA during winter suggests that cold stress induced significant oxidative stress in camels. Cold exposure is known to elevate metabolic activity and reactive oxygen species (ROS) production, leading to enhanced lipid peroxidation, as reflected by increased MDA levels. In response, animals upregulate antioxidant enzymes such as CAT as a compensatory mechanism to counteract oxidative damage [18]. However, the concurrent elevation of both CAT and MDA indicates that the antioxidant defense system was insufficient to fully neutralize ROS, resulting in oxidative imbalance. TAC was higher in autumn compared to both summer and winter, suggesting that autumn represents a thermoneutral condition with minimal environmental stress. In contrast, both heat and cold stress conditions appear to deplete antioxidant reserves, leading to reduced TAC. The more pronounced oxidative stress response observed during winter compared to summer may be attributed to the superior heat tolerance of camels, which are well adapted to hot environments but less adapted to cold stress. The mean levels of dromedary camel hormones (T3, T4, and cortisol) in the present study were in line with previous reports [10, 19]. T3 showed a significant increase, while T4 showed a non-significant increase during winter compared to summer and autumn, in agreement with earlier studies [10, 20, 21]. Cold environment may be a stimulus to increase the output of thyrotrophic hormone, thereby resulting in a higher concentration of thyroid hormones in serum in winter [20]. Thyroid hormones play a crucial role in thermogenesis by increasing basal metabolic rate, oxygen consumption, and mitochondrial activity, thereby helping animals maintain body temperature under cold conditions [22]. During summer, serum concentrations of T3 and T4 reduced and were likely to be due to the direct effect of heat stress on the hypothalamic- pituitary—thyroid (HPT) axis to decrease the production of thyrotropin- releasing hormone, which will limit basal metabolism which may suggest a physiological adaptation to heat stress, where suppression of thyroid activity helps minimize endogenous heat production and preserve body water in dromedary camel [23]. Cortisol levels showed a significant increase in both summer and winter compared with autumn, in agreement with [20]. The observed increase in cortisol levels during both summer and winter suggests activation of the hypothalamic–pituitary–adrenal (HPA) axis in response to environmental stress [23]. Both heat and cold stress are known to stimulate cortisol secretion as part of the adaptive stress response. In contrast, the lower cortisol levels observed during autumn indicate that this season likely represents a thermoneutral condition with minimal environmental stress. metabolic profile levels observed in this study were consistent with those reported in previous studies [19, 24]. Glucose levels showed a significant increase during winter, in agreement with [25], whereas triglyceride levels were significantly higher in autumn compared to summer and winter, consistent with the findings of [20]. The increased glucose levels in winter could be likely due to enhanced gluconeogenesis driven by elevated cortisol and thyroid hormones under cold stress. In contrast, triglycerides were highest in autumn, reflecting reduced energy utilization under thermoneutral conditions. Zinc and copper levels observed in this study were consistent with those reported in previous studies [26–28]. Despite the higher oxidative stress observed in winter, zinc and copper levels were elevated in summer in agreement with [29]. This pattern suggests that their increase is more likely related to hemoconcentration resulting from dehydration under heat stress. In contrast, the lower levels of these trace elements during winter may be explained by increased utilization and redistribution from the circulation to tissues in response to heightened oxidative stress. Zinc and copper are essential cofactors for antioxidant enzymes [11], and their increased demand under cold stress conditions may reduce their circulating levels. These findings indicate that seasonal stress in camels influences multiple physiological systems, with cold stress exerting a more pronounced effect on oxidative balance and metabolic activity, while heat stress primarily affects fluid balance and circulating trace element concentrations.

The strong positive correlation observed between T3 and T4, consistent with a previous report [9], suggests coordinated regulation of thyroid hormone secretion through activation of the HPT axis. Since T4 serves as the primary precursor for T3, increased T4 availability enhances peripheral conversion to T3, thereby reinforcing their positive association. This relationship is further supported by the concurrent elevation of both hormones during winter, indicating that seasonal stress, particularly cold exposure, stimulates overall thyroid activity. The very strong negative correlation observed between zinc and cholesterol may reflect distinct physiological regulation of zinc homeostasis and cholesterol metabolism. Zinc is known to influence hepatic lipid metabolism and affect the expression of genes encoding enzymes contributing to liver lipid homeostasis [30, 31]. Lowered zinc levels during winter may result from increased utilization and redistribution to support antioxidant defense mechanisms under heightened oxidative stress. In contrast, cholesterol levels remained relatively stable across seasons. These differences in responsiveness may contribute to the observed inverse relationship between zinc and cholesterol. The positive correlation between glucose and MDA was consistent with [7] and suggests a close association between energy metabolism and oxidative stress. Elevated glucose levels can enhance ROS production through increased mitochondrial activity and activation of oxidative pathways, leading to lipid peroxidation as reflected by increased MDA levels [32]. The positive correlation between cortisol and CAT suggests a coordinated physiological response to stress. Increased CAT activity reflects the upregulation of antioxidant defenses in response to enhanced ROS production [7]. Cortisol may contribute indirectly to oxidative stress through increased metabolic activity and gluconeogenesis, thereby promoting ROS generation. In turn, the body enhances antioxidant enzyme activity, including CAT, as a compensatory mechanism to mitigate oxidative damage. This relationship is consistent with the observed seasonal pattern, where both cortisol and oxidative stress biomarkers were elevated during winter, suggesting that cold stress induces a coupled endocrine and oxidative response. The positive correlation between copper and TAC suggests the essential role of copper in antioxidant defense mechanisms. Copper serves as an essential cofactor for several antioxidant enzymes, including superoxide dismutase, which contributes to the neutralization of reactive oxygen species [9]. Additionally, copper is associated with ceruloplasmin, a protein with antioxidant properties that may significantly contribute to overall antioxidant capacity. Since TAC represents the cumulative effect of both enzymatic and non-enzymatic antioxidants, higher copper levels may be indicative of enhanced antioxidant potential. The negative correlation between triglycerides and CAT, T4, and cortisol reflects the metabolic shift from energy storage to energy utilization under stress conditions. Triglycerides represent a major form of energy storage, whereas elevated cortisol and thyroid hormones promote lipolysis and increase metabolic rate, leading to enhanced lipid utilization [10, 20]. Similarly, increased CAT activity indicates heightened oxidative stress, which is often associated with increased metabolic demand and energy consumption. Under such conditions, triglyceride levels decrease as they are mobilized to meet energy requirements. These findings suggest that seasonal stress shifts the metabolic balance toward energy mobilization, resulting in an inverse relationship between triglycerides and stress-related physiological parameters. The negative correlation between zinc and triglycerides may be attributed to the role of zinc in regulating lipid metabolism and its involvement in stress adaptation [31]. Conversely, the higher triglyceride concentrations observed during autumn may suggest reduced metabolic demand and lower zinc availability, supporting an inverse relationship between these parameters. The negative correlation between glucose and cholesterol may be attributed to the fact that glucose serves as a rapidly responsive energy substrate under stress, whereas cholesterol is more tightly regulated, with its levels remaining relatively stable during heat and cold seasons, thereby exhibiting an inverse relationship with glucose during periods of increased metabolic demand. The negative correlation between copper and MDA suggests a protective role of copper against oxidative stress. Accordingly, higher copper concentrations were associated with reduced lipid peroxidation, as indicated by lower MDA levels.

Seasonal correlation analysis identified several associations that were not evident in the pooled analysis, indicating that environmental temperature may modify the interactions among thyroid hormones, oxidative stress biomarkers, minerals and lipids. During the summer season, the correlation between T3 and T4 and glucose and malondialdehyde were consistent with the overall correlation analysis discussed previously. The negative correlations between glucose and both T3 and T4 may reflect metabolic adaptation to heat stress and could be related to the role of thyroid hormones in pancreatic β-cell development and glucose metabolism through multiple organs, including the pancreas, gastrointestinal tract, liver, and central nervous system. In addition, T3 has been shown to regulate hepatic glucose production through a sympathetic pathway connecting the hypothalamic paraventricular nucleus (PVN) and the liver. Increased T3 activity within the hypothalamic PVN stimulates hepatic glucose production independently of glucoregulatory hormones [33]. The positive correlation between cholesterol and thyroid hormones observed during summer could be linked to their similar seasonal pattern, as both parameters reached their lowest values during this season. This association is more likely to represent a common physiological response to heat stress rather than a direct regulatory effect of thyroid hormones on cholesterol metabolism. The negative correlation between zinc and copper during summer may reflect the well-established antagonistic interaction between these trace elements, as Zn^2^⁺ and Cu^2^⁺ share the same primary binding site, allowing Zn^2^⁺ to compete with Cu^2^⁺ for cellular uptake [34]. During autumn, the correlations between T3 and T4, glucose and cholesterol, and zinc and cholesterol were consistent with those identified in the overall correlation analysis and have been discussed previously. The positive correlation between CAT activity and TAC indicates coordinated activation of the antioxidant defense system. CAT is one of the major enzymatic antioxidants responsible for hydrogen peroxide detoxification, whereas TAC reflects the overall antioxidant capacity of biological fluids. Their positive association suggests that enzymatic and non-enzymatic antioxidant mechanisms act synergistically to maintain redox homeostasis under normal environmental conditions. The negative correlation between triglycerides and TAC may be explained by increased utilization of antioxidant defenses under conditions associated with altered lipid metabolism. The negative correlation between cortisol and T4 may reflect the inhibitory effect of cortisol on the secretion of thyrotropin-releasing hormone (TRH) by the hypothalamus and thyroid-stimulating hormone (TSH) by the pituitary gland, thereby reducing thyroid hormone synthesis [35]. The positive correlation between zinc and glucose observed during autumn may represent the physiological role of zinc in glucose homeostasis under thermoneutral conditions. Zinc has been shown to facilitate cellular glucose uptake through the phosphoinositide 3-kinase (PI3K) signaling pathway and exhibits insulin-like actions that enhance glucose transport and lipogenesis [36]. During winter, the strong negative correlation between T3 and TAC suggests that enhanced thyroid activity during cold exposure may increase metabolic rate and mitochondrial oxygen consumption, leading to greater production of reactive oxygen species and increased utilization of antioxidant defenses. Consequently, higher circulating T3 concentrations may be associated with lower TAC values under cold stress. The positive correlation between T3 and zinc observed during winter is biologically plausible because zinc is essential for thyroid hormone synthesis, peripheral conversion of T4 to T3, and the maintenance of tissue responsiveness to thyroid hormones [37]. adequate zinc availability may therefore support enhanced thyroid activity required for thermogenesis during cold adaptation.

Since cold stress was associated with higher oxidative stress and metabolic burden, it is recommended to provide adequate shelter, wind protection, and appropriate bedding during winter to minimize heat loss and reduce physiological stress. Dietary strategies during cold season should focus on increasing energy density and incorporating antioxidant-rich supplements (e.g., vitamin E, selenium, zinc, and copper) to counteract oxidative stress and support metabolic demands.

### Study limitations

A limitation of the present study is that the same camels were not consistently available throughout all sampling seasons because of routine herd turnover, including animal replacement and slaughter in the participating farms. To minimize potential confounding, animals were selected from the same two farms within the Greater Cairo area and were matched as closely as possible for age, sex, body weight, feeding regimen, and management conditions. Therefore, the findings should be interpreted as herd-level seasonal comparisons rather than longitudinal changes within the same individuals. Future longitudinal studies following the same animals across different seasons are warranted to validate the present findings.

## Conclusion

Winter was associated with the highest oxidative stress, as indicated by increased MDA and CAT activity and decreased TAC, suggesting that cold stress imposes a greater oxidative burden than heat stress. This was accompanied by increased T3, T4, and cortisol levels, and decreased triglycerides and copper levels, suggesting an integrated physiological response aimed at increasing metabolic rate and energy mobilization to cope with cold stress. Correlation analysis revealed significant associations among endocrine, oxidative, and metabolic parameters. The observed seasonal differences suggest that environmental temperature modifies these associations. Overall, cold stress exerted a greater impact on oxidative balance and metabolism in camels than heat stress. Therefore, ensuring adequate dietary zinc and copper supply during winter and regularly monitoring oxidative, hormonal, mineral, and metabolic parameters are recommended to support health and enable early intervention.

## Data Availability

All data generated or analysed during this study are included in this published article.

## Abbreviations

MDA: Malondialdehyde
CAT: Catalase
TAC: Total antioxidant capacity
T3: Triiodothyronine
T4: Thyroxine
ROS: Reactive oxygen species
SOD: Superoxide dismutase
GPx: Glutathione peroxidase
Cu/Zn-SOD: Copper-zinc superoxide dismutase
T: Ambient temperature
RH: Relative humidity
THI: The temperature–humidity index
SPSS: Statistical Package for Social Sciences
ANOVA: Analysis of variance
HSD: Honestly significant difference
SE: Standard error
HPT: Hypothalamic–pituitary–thyroid axis
HPA: Hypothalamic–pituitary–adrenal axis
PVN: Paraventricular nucleus
TRH: Thyrotropin-releasing hormone
TSH: Thyroid-stimulating hormone
